# Performance of Physical Examination Skills in Medical Students during Diagnostic Medicine Course in a University Hospital of Northwest China

**DOI:** 10.1371/journal.pone.0109294

**Published:** 2014-10-15

**Authors:** Yan Li, Na Li, Qunying Han, Shuixiang He, Ricard S. Bae, Zhengwen Liu, Yi Lv, Bingyin Shi

**Affiliations:** First Affiliated Hospital, School of Medicine, Xi'an Jiaotong University, Shaanxi, Xi'an, China; University College London, United Kingdom

## Abstract

This study was conducted to evaluate the performance of physical examination (PE) skills during our diagnostic medicine course and analyze the characteristics of the data collected to provide information for practical guidance to improve the quality of teaching. Seventy-two fourth-year medical students were enrolled in the study. All received an assessment of PE skills after receiving a 17-week formal training course and systematic teaching. Their performance was evaluated and recorded in detail using a checklist, which included 5 aspects of PE skills: examination techniques, communication and care skills, content items, appropriateness of examination sequence, and time taken. Error frequency and type were designated as the assessment parameters in the survey. The results showed that the distribution and the percentage in examination errors between male and female students and among the different body parts examined were significantly different (*p*<0.001). The average error frequency per student in females (0.875) was lower than in males (1.375) although the difference was not statistically significant (*p* = 0.167). The average error frequency per student in cardiac (1.267) and pulmonary (1.389) examinations was higher than in abdominal (0.867) and head, neck and nervous system examinations (0.917). Female students had a lower average error frequency than males in cardiac examinations (*p* = 0.041). Additionally, error in examination techniques was the highest type of error among the 5 aspects of PE skills irrespective of participant gender and assessment content (*p*<0.001). These data suggest that PE skills in cardiac and pulmonary examinations and examination techniques may be included in the main focus of improving the teaching of diagnostics in these medical students.

## Introduction

Diagnostic medicine plays an important role in bridging basic medicine and clinical medicine [1], [2]. The content of diagnostics includes inquiry, physical examination, laboratory examination and other ancillary examinations. The abilities acquired with regards to diagnostics accompany a doctor through his or her entire career, from medical school to internship to clinical practice.

Physical examination (PE) skills are basic and essential elements of clinical competency for medical staff [3]. In PE, one of the four clinical diagnostic methods, physicians use their senses and traditional tools, such as thermometer, sphygmomanometer, percussion hammer, and stethoscope, to objectively understand and systematically assess the patient, and discover normal and abnormal signs. PE skills are the mainstay of clinical diagnosis in rural hospitals, where physical and financial access to other tests is extremely limited. Performing a proper physical examination using four modalities (inspection, palpation, percussion and auscultation) also provides the physical contact that communicates a doctor's caring touch to the patient. In a recent study, performance of the general physical examination was shown to be already below expectation at the end of the internal medicine clerkship [4]. However, no grave concern over medical students' performance of PE skills during their diagnostic medicine course has been addressed.

Chinese medical students are required to systematically learn PE skills for the first time during their diagnostic medicine course. The objective of this study was to conduct an investigation on the performance of PE skills during their diagnostic medicine course and analyze the characteristics of the data collected to provide information for practical guidance to improve the quality of teaching.

## Methods

### Ethics Statement

This study was approved by the Institutional Research Ethics Board of the First Affiliated Hospital, School of Medicine, Xi'an Jiaotong University. All participants provided their written informed consent to participate in this study and this consent procedure was approved by the Institutional Research Ethics Board.

### Data collection

This study was conducted in a university hospital in northwest China - the First Affiliated Hospital, School of Medicine, Xi'an Jiaotong University. This hospital is a typical institution for clinical medical education in China and the biggest hospital directly under the administration of the Chinese Ministry of Health in northwest China. The hospital also is the site of “The National Clinical Skills Training Center”. Our school is one of the 15 key medical colleges and universities and one of the earliest institutions qualified for the seven-year medical school program in China. Xi'an Jiaotong University is a key national university under the direct jurisdiction of the Ministry of Education of China, which, through the national university entrance examination, enrolls all students nationwide, including medical students, excluding Taiwan, Hong Kong and Macau. The standards of medical education and the detailed rules in the form of government documents and the means of evaluation or authentication were formulated by the Ministry of Health and the Ministry of Education of China. Diagnostic medicine is included in the curriculum of clinical medicine, and physical examination (PE) is one of the clinical skills that the medical students must master. The students used a textbook of diagnostic medicine compiled for universal use of the 7-year medical school program of universities in China.

To evaluate the representativeness of our students, we compared the demographic variables of our students with those of the medical students enrolled at the same year (2008) for 7-year medical school program in other 2 universities: Harbin Medical University and Anhui Medical University with permission of these 2 universities.

From our 7-year medical school program, all the 72 fourth-year medical students, 32 males and 40 females, taking their diagnostic medicine course were included in our study. The average age of participants was 19–25 (21.2±1.3) years. After 17 weeks of formal training and systematic teaching, all participants received an assessment of PE skills as part of their diagnostic medicine final examination at the end of the fall semester in the 2011–2012 school year.

The assessment content was randomly assigned to each student, covering all of the PE procedures and skills (i.e. inspection, palpation, percussion and auscultation), as well as complete body parts (i.e. head and neck, pulmonary, cardiac, abdominal, nervous system). Male students acting as patients were used. Each student had one patient encounter. We used the General Physical Examination Assessment Standards of Xi'an Jiaotong University (Table S1), which was enacted by the Xi'an Jiaotong University Clinical Teaching Committee.

Students' performance of PE skills was evaluated and recorded in detail using a well-designed checklist, including 5 aspects of PE skills: examination techniques, communication and care skills, content items, appropriateness of examination sequence, and time taken (Table S2). The error frequency and type were designated as the assessment parameters in the survey. In order to ensure objectivity and fairness in the survey, each student had two teachers evaluating and scoring at the same time. The score was accepted for a student if the two teachers had the same scoring results. If a student received two different scores, a final single score was given only after the two teachers reached a consensus. In fact, the concidence rates of scoring results by two teachers was high with only 2 instances of inconsistency in the whole evaluation (1 time in pulmonary percussion in 1 student and 1 time in abdominal palpation in another student). The consensus results were readily achieved by the two teachers. The participating teachers were told the evaluation method and intent of each assessment parameter and were trained to properly apply the checklist derived from the “General Physical Examination Assessment Standards of Xi'an Jiaotong University” (Table S1). Prior to the actual evaluation, the teachers underwent a one-week training about the evaluation and performed simulated evaluations, which included scoring and filling out the evaluation checklist. The teachers were all senior physicians at the university hospital, with experience in clinical teaching and patient management. All the students were evaluated and recorded by the same two teachers.

### Statistical analysis

After completion of the assessment, the surveyed data were collected and expressed as the mean ± SD or frequency. Statistical analysis was performed using SPSS software version 16.0 (SPSS, Inc., Chicago, IL). Rank and categorical variables were compared between groups, using the Wilcoxon rank sum test or the Chi-square test where appropriate. A *P* value <0.05 was considered statistically significant.

## Results

### Comparison of the demographics of the students

Age and ethnicity were similar (no significant difference) between the students from the two universities and the language was also similar. The gender distribution of our students was also not siginificantly different from that of the students from the other 2 universities although it is significant between the students from the other 2 universities. The demographics and source regions of the medical students in the 3 universities are shown in Table S3.

### Correlations of error frequency of PE skills

Error frequencies that students made in PE skills were recorded as 6 categories: 0, 1, 2, 3, 4 and 5. There was a statistically significant difference between the distribution and the percentage in error frequency when separated by gender (*p*<0.001, Table 1). First, the highest error frequency in the male group was 4 and that in the female group was 2. Second, the most common error frequency in the male group was 2 with 34.4% (11/32) having an error frequency of 2 and in the female group was 1 [42.5% (17/40)]. In total, no student had 5 error frequencies of PE skills in the survey, and the most common error frequency was 1, accounting for 36.1% (26/72).

**Table 1:** Error frequency of five error types in the students.

Participants	Average error frequency per student	Error frequency of physical examination skills [n (%)]					
		0	1	2	3	4	5
Male (n = 32)	1.375 (44/32)	8 (25.0%)	9 (28.1%)	11 (34.4%)	3 (9.4%)	1 (3.1%)	0 (0%)
Female (n = 40)	0.875 (35/40)	14 (35.0%)	17 (42.5%)	9 (22.5%)	0 (0%)	0 (0%)	0 (0%)
Total (n = 72)	1.097 (79/72)	22 (30.6%)	26 (36.1%)	20 (27.8%)	3 (4.2%)	1 (1.4%)	0 (0%)

Average error frequency per student: Pearson chi square, *p* = 0.167; Error frequency of physical examination skills: Wilcoxon rank sum test, *p*<0.001.

In order to directly compare the differences, we introduced a new index, the average error frequency per student, which divided total error frequency by the number of participants. In this survey, the error frequency per student in females (0.875) was less than that in males (1.375) although it did not reach statistical significance, perhaps because of small sample size (*p* = 0.167, Table 1).

We further performed statistical analysis on the error frequency in assessment content according to the body parts examined. In this survey, the assessment content was randomly assigned to each student, covering all of the PE skills and all of the body parts. To balance the assessment content, we combined head, neck and nervous system into one group. Then we formed four groups for the purpose of analysis, namely, head, neck and nervous system, cardiac, pulmonary, and abdominal.

There was a significant difference between the distribution and the percentage in error frequency of the different body parts examined (*p*<0.001, Table 2). The highest error frequency of pulmonary examination was 4, head neck and nervous system examination and cardiac examination were 3, and abdominal examination was 2. The most common error frequency of cardiac examination was 0 and 2 (both 33.3%) and that of pulmonary examination was 2 (44.4%). The most common error frequency of head, neck and nervous system was 1 (45.8%) and abdominal was 1 (46.7%).

**Table 2:** Error frequency in the students according to body parts examined.

Body parts examined	Average error frequency per student	Error frequency of physical examination skills [n (%)]					
		0	1	2	3	4	5
Head, neck and nervous system (n = 24)	0.917(22/24)	8 (33.3%)	11 (45.8%)	4 (16.7%)	1 (4.2%)	0 (0%)	0 (0%)
Cardiac (n = 15)	1.267(19/15)	5 (33.3%)	3 (20.0%)	5 (33.3%)	2 (13.3%)	0 (0%)	0 (0%)
Pulmonary (n = 18)	1.389(25/18)	4 (22.2%)	5 (27.8%)	8 (44.4%)	0 (0%)	1 (5.6%)	0 (0%)
Abdominal (n = 15)	0.867(13/15)	5 (33.3%)	7 (46.7%)	3 (20.0%)	0 (0%)	0 (0%)	0 (0%)
Total (n = 72)	1.097(79/72)	22 (30.6%)	26 (36.1%)	20 (27.8%)	3 (4.2%)	1 (1.4%)	0 (0%)

Average error frequency per student: Pearson chi square, *p* = 0.678. Error frequency of physical examination skills: Wilcoxon rank sum test, *p*<0.001.

The average error frequency per student in cardiac and pulmonary examinations (1.267 and 1.389, respectively) was higher than that in abdominal examination (0.867) and head, neck and nervous system examinations (0.917) (*p* = 0.678, Table 2). To further look at the differences found between the cardiac and pulmonary examinations, we analyzed the data based on gender and PE skills (inspection, palpation, percussion and auscultation).

The average error frequency per student in females was less than that in males in cardiac examination (*p* = 0.041, Table 3). Another finding was that the error frequency in PE skills of cardiac examination (i.e. inspection, palpation, percussion and auscultation) according to gender is statistically different (*p* = 0.009, Table 4). Inspection and palpation in cardiac examination were more error-prone in male than female students, although the differences were only marginally significant (both *p* = 0.054), and percussion in cardiac examination was more error-prone in female students (*p* = 0.008, Table 4).

**Table 3:** Error frequency per student in cardiac and pulmonary examinations.

Examination	Participants	Total*	Error frequency per student in each assessment			
			Inspection	Palpation	Percussion	Auscultation
Cardiac	Male	2.13 (17/8)	3 (6/2)	3 (6/2)	1 (2/2)	1.5 (3/2)
	Female	0.80 (8/10)	0 (0/1)	0 (0/1)	1.25 (5/4)	0.75 (3/4)
	Total	1.39 (25/18)	2 (6/3)	2 (6/3)	1.17 (7/6)	1 (6/6)
Pulmonary	Male	1.63 (13/8)	2 (2/1)	2 (2/1)	2 (6/3)	1 (3/3)
	Female	1.15 (15/13)	0.5 (1/2)	0.5 (1/2)	1.5 (9/6)	1.33 (4/3)
	Total	1.33 (28/21)	1 (3/3)	1 (3/3)	1.67 (15/9)	1.17 (7/6)

*Data are calculated as total error frequency/students (n). Comparison of gender in cardiac examination: Wilcoxon method, *p* = 0.041, Comparison of gender in pulmonary examination: Wilcoxon method, *p* = 0.074.

**Table 4:** Error frequency in physical examination skills of cardiac and pulmonary examinations.

Examination skills	Cardiac examination			Pulmonary examination		
	Male	Female	*p*	Male	Female	*p*
Inspection	6 (35.3%)	0 (0%)	0.054	2 (15.4%)	1 (6.7%)	0.457
Palpation	6 (35.3%)	0(0%)	0.054	2 (15.4%)	1 (6.7%)	0.457
Percussion	2 (11.8%)	5 (62.5%)	0.008	6 (46.1%)	9 (60.0%)	0.464
Auscultation	3 (17.6%)	3 (37.5%)	0.278	3 (23.1%)	4 (26.6%)	0.827
Total	17(100%)	8 (100%)		13 (100%)	15 (100%)	

Data are calculated as error frequency/total error frequency (n). Comparison of cardiac examination: Pearson chi square, *p* = 0.009. Comparison of pulmonary examination: Pearson chi square, *p* = 0.736.

The error frequency in pulmonary examination in male students was higher than that in female although the difference was not statistically significant (*p* = 0.074, Table 3). Further analysis showed that percussion of pulmonary examination was the most error-prone point both in female and male students with no significant difference between genders. The error frequency of PE skills (inspection, palpation, percussion and auscultation) in pulmonary examination was not statistically different between females and males (*p* = 0.736, Table 4).

### Correlations of error types of PE skills

The error frequency that students made in PE skills was recorded according to the five categories: examination techniques, communication and care skills, content items, appropriateness of examination sequence, and time taken. We found that error in examination technique was the highest error type among the 5 aspects of PE skills (*p*<0.001, Table 5), with no relationship to participant gender and body parts examined (*p* = 0.405; *p* = 0.367, Table 5). Communication and care skills errors were the second highest error type among the 5 aspects of PE skills (Table 5) although comparison of communication and care skills with items, appropriateness of physical examination sequence, and time taken showed no significant difference.

**Table 5:** Error types in the students and error types according to body parts examined.

	Total error	Types of physical examination skills				
		Techniques	Communication and care skills	items	appropriateness of PE sequence	time taken
Head, neck and nervous system	22	14 (63.6%)	1 (4.5%)	2 (9.1%)	4 (18.2%)	1 (4.5%)
Cardiac	19	8 (42.1%)	4 (21.0%)	3 (15.8%)	1 (5.3%)	3 (15.8%)
Pulmonary	25	9 (36.0%)	7 (28.0%)	4 (16.0%)	3 (12.0%)	2 (8.0%)
Abdominal	13	9 (69.2%)	1 (7.7%)	2 (15.4%)	0 (0%)	1 (7.7%)
Total	79	40 (50.6%)	13 (16.5%)	11 (13.9%)	8 (10.1%)	7 (8.9%)
Male	44	19 (43.2%)	9 (20.5%)	7 (15.9%)	6 (13.6%)	3 (6.8%)
Female	35	21 (60.0%)	4 (11.4%)	4 (11.4%)	2 (5.7%)	4 (11.4%)

Data are calculated as error frequency/total error frequency (n). Comparison in gender: Pearson chi square, *p* = 0.405. Comparison of techniques with communication and care skills, items, appropriateness of physical examination sequence and time taken in pairwise: Pearson chi square, *p* = 0.001, <0.001, <0.001 and <0.001, respectively. Comparison among assessment content (body parts), Pearson chi square *p* = 0.367.

## Discussion

Studies of physical examination skills in U.S. medical students have consistently identified deficiencies in physical examination performance among third- and fourth-year medical students, and received much attention from global medical educators [5]–[9]. Though there is a large difference in the modes of education, curricula, course content, and teaching methodologies between Chinese and non-Chinese medical education systems, it is commonly agreed upon that physical examination skills are basic and essential elements of clinical competency for all medical staff. In this study, we empirically classified error types of PE skills into five types: techniques, communication and care skills, content items, appropriateness of examination sequence, and time taken. We then evaluated the performance of PE skills in the medical students at the end of their diagnostic medicine course.

Although we failed to obtain the national data, the demographic variables of our students had no significant differences in the age, gender, ethnicity and language with those of the medical students enrolled at the same year for the same medical school program in other 2 universities, indicating the representativeness of our students to some extent.

According to our findings in error frequencies of PE skills, there was a gender difference, with the performance of female students being better than that of male students. Skills of inspection and palpation in cardiac examination appeared to be more error-prone in male than female students and skills of percussion in cardiac examination were more error-prone in female students. One study showed that anxiety and gender had influence on both self-assessment and actual performance on high-stakes clinical skills, such as history taking and physical examination [10]. The females with high anxiety usually outperform the males with high anxiety on self-assessment accuracy and actual clinical performance [10]. In contrast, a previous study indicated that there were no gender differences in the majority of the tasks performed in a family medicine clerkship [11]. Differences were only found in gender-specific procedures in their survey, showing that breast and pelvic examinations were more frequently performed by female students, and testicular examination performed better by males [11]. Interestingly, some gender-related qualities may be associated with the difference in the performance of physical examination skills. For instance, the verbal ability in girls was shown to be higher than boys [12]–[14] and this may benefit girls in their communication and cooperation with the patients during physical examination. In fact, the types of physical examination skills of our study showed that female students had a significantly lower error frequency in communication and care skills than male students. In addition, prepubescent girls, in comparison with boys, were found to be more likely to have superior manual control abilities for performing novel tasks [15]. Females were also found to be more sensitive than males in fingertip recognition of micron-level randomness as unpleasant [16] and to perform better than males in the haptic change task [17]. These characteristics in females may also contribute, at least to some extent and in some aspect, to the better performance of their physical examination. Moreover, the differences in acculturation to medicine and feelings of entitlement between male and female, although the possible involvements need to be investigated, may also incur some difference in performing physical examination between male and female. Further empirical data are needed to determine if the gender difference with regards to PE skills in this study can be generalized to other clerkships or internships.

Data from our survey showed a statistical difference in the distribution of error frequency of PE skills in the different body parts examined, with errors in cardiac and pulmonary examinations being the most common. This is consistent with our teaching experience. Different from the findings in the present study, some literature reported that the five subcomponents of the complete physical examination (head, neck, ear-nose-throat; lungs, thorax, breast, abdomen; heart, pulses, vitals; musculoskeletal; neurologic) were different in difficulty and reliability, and the average percent correct of lungs, thorax, breast, abdomen and neurologic examination was below the average percent correct of total examination and the average percent correct for cardiac was above the average percent correct of total examination [18]. In a different study which included abdominal, cardiac, pulmonary, and vascular but not neurologic assessment, it was found that the mean percent correct scores of cardiac and vascular were below that of total [19]. Other investigators demonstrated similar shortcomings in residents' examination skills, particularly in the cardiovascular examination [20]. It is suggested that PE skills in cardiac and pulmonary examinations are among the areas that the teachers should pay more attention and that the teaching methodology in cardiac and pulmonary examinations needs further improvement. It has proven useful to utilize instructional videos on standard procedure of physical examination. Medical students can use such videos for independent learning, while clinical teachers can adopt them as teaching resources. In addition to the standard procedure, it will be much more helpful to also address common PE mistakes in such videos [21], [22].

Another finding in this study was that error in technique was the highest error type among the five aspects of PE skills, with no relationship to student gender or assessment content. Previous investigation found that the five most commonly missed items were inspection of the skin, complete examination in logical sequence, palpation of the aorta, auscultation of anterior breath sounds, and palpation of axillary and inguinal nodes. Other important observed errors were failure to measure vital signs, incorrect identification of liver and spleen, failure to use the bell on the stethoscope, and an inadequate breast examination [20]. It is suggested that technique error should be included in the focus of improving the teaching of diagnostics. For medical students at the beginning of clinical clerkships, techniques of PE would be a challenge and may only be mastered well by long-term practice. It is revealed by previous research that near-peers can be effective teachers in preclinical courses and clinical clerkship, e.g., anatomy and physical examination [23]. The authors found that the students can more effectively learn physical examination skills by studying within a group than by studying individually. When studying in a group, students have the chance to observe and learn from others during practice, which helps them to acquire the physical examination skills better [3], [24]. Simulated and real patients strongly inspire students to work on skills. Through early contact with patients, students can feel more prepared when carrying out their clinical practice. Simulation training and feedback may have some influence on skills outcomes [25], [26].

The lack of efficient communication and care skills is another issue in current Chinese clinical education. Frequency of communication and care skills errors is among the second highest error type of PE skills in our study. A similar phenomenon has also been noticed in literature. For example, senior medical students were found to rarely communicate with patients during the physical examination [27]. Standard patient-narrated web-based learning modules appear to be useful in enhancing the students' communication skills on high-stakes clinical skills examination [28]. In order to make an effective plan to teach medical students specific communication skills when carrying out the physical examinations, the initial step is to find out how the students communicate with patients without specific training. Furthermore, we need to identify how to explore communication during physical examination, as well as how to teach and learn such communication skills.

There are limitations to our study. The number of students evaluated is small and the definition of the evaluation content and criteria are primarily empirical. The scoring system and the training of the teachers who performed the evaluation was not standardized. The data analyzed were obtained from only one grade of medical students in a single institution. Additional studies are needed to confirm the validity and reliability of the assessment instrument and standardize the evaluation system. Furthermore, a multivariate analysis may provide more information and convincing evidence regarding the performance of PE skills in medical students. However, we did not conduct multivariate analysis because that the sample size is too small. The failure to obtain national data of the students and to perform corresponding comparisons may affect the representativeness of our study. Therefore, caution should be taken in interpreting and expanding our findings and further studies in multiple grades of students and more institutions with multivariate analysis are needed to confirm our findings.

### Conclusions

This survey showed that PE skills in cardiac and pulmonary examinations and technique in PE are among the more common error-prone areas in the medical students of our university. More attention and innovation in teaching methodology associated with these contents should be included in the focus of improving the teaching of diagnostic medicine. Further data are needed to determine whether gender differences with regards to PE skills suggested in this study are important and if they can be generalized to other clerkships or internships.

## Supporting Information

Table S1 General Physical Examination Assessment Standards of Xi'an Jiaotong University.
(DOC)

Table S2 Evaluation checklist of students' PE skills.
(DOC)

Table S3 Demographics and source regions of the medical students enrolled in 2008 in three universities of China.
(DOC)
