# Supplementary material for: Performance of Physical Examination Skills in Medical Students during Diagnostic Medicine Course in a University Hospital of Northwest China
Source: PLoS One. 2014 Oct 15;9(10):e109294. doi: 10.1371/journal.pone.0109294 (PMC4198092; doi:10.1371/journal.pone.0109294)
Supplement: Table S1 — General Physical Examination Assessment Standards of Xi'an Jiaotong University. (DOC) [file pone.0109294.s001.doc]

**Table S1 General Physical Examination Assessment Standards of Xi’an Jiaotong University**

**A. GENERAL EXAMINATION AND VITAL SIGNS**

1. First, arrange all physical examination instruments properly. Then, standing at the right side of the patient, after taking the medical history, tell the patient the elements of the physical exam.

2. Measure the axillary temperature. Place the thermometer under the patient’s armpit directly in contact with the skin.

3. Palpate the radial pulse for at least 30 seconds.

4. Observe the respiratory rate for 30 seconds.

5. Measure the blood pressure on the right arm. Place the cuff so that the lower edge is 2-3 cm above the antecubital fossa and place the stethoscope firmly over the brachial artery at the level of the mid-axillary line; then observe the liquid mercury column at eye level. Measure the blood pressure twice, waiting a minute or so between readings. After finishing the measurement, lean the manometer over a little so the mercury column disappears, close the mercury column switch, and close the manometer.

6. Remove the thermometer, observe the reading, and shake down the mercury.

7. Observe the following aspects: development, nutrition, body habitus, facial features and expression, and body posture.

**B. HEAD**

8. Observe the appearance of the hair and skull.

9. Palpate the skull.

10. Observe the eyelids. After everting the upper eyelids, observe the upper and lower palpebral conjunctiva, conjunctival fornix, and bulbar conjunctiva and sclera, first left then right.

11. Observe the shape of the eyeballs and pupils.

12. Using a penlight, test bilateral direct and indirect pupillary light reflexes.

13. Evaluate bilateral extraocular muscle function by having the patient follow your index finger from the center to six directions: left, upper left, and lower left, right, upper right, lower right.

14. Check the accommodation reflex.

15. Check for convergence. Do not check for the corneal reflex in conscious patients.

16. From left to right, examine the bilateral pinnae, external auditory canals, and mastoid processes.

17. Inspect the external nose, nasal vestibule and nasal cavity, examine bilateral nasal passages.

18. Palpate and percuss the frontal, ethmoid, and maxillary sinuses bilaterally.

19. Observe the lips; the examiner may use a sterile tongue depressor to aid inspection of the buccal mucosa, teeth, gums, tonsils and posterior oropharyngeal wall, etc.; inspect the tongue for its shape, coating, and movement (tongue protrusion, show teeth, and puff out cheeks).

**C. NECK**

20. Examine the skin and blood vessels of the neck from left to right, and observe the thyroid.

21. Palpate the lymph nodes of the neck in the following order: preauricular nodes, postauricular nodes, mastoid region, occipital nodes, posterior cervical nodes, anterior cervical nodes, supraclavicular nodes.

Posterior cervical lymph nodes: use the fingertips of both hands to palpate between the trapezius muscle and the sternocleidomastoid muscle

Anterior cervical lymph nodes: with palms facing up, palpate using both hands along the border of the sternocleidomastoid muscle, then use the right hand on the left side to move along the left jaw towards the chin palpating the submental nodes, and do the same for the other side.

Supraclavicular nodes: with the patient leaning forward, use the fingertips of both hands to palpate along the upper border of the clavicles.

22. Palpate the isthmus and lobes of the thyroid gland. Place the right thumb on the suprasternal notch and palpate as the patient swallows; place the left thumb under the thyroid cartilage, gently pushing from the right side to the opposite side, with the right index, middle, and ring fingers on the left edge of the sternocleidomastoid muscle and the right thumb moving along the side of the trachea palpating the left lobe of the thyroid gland while the patient swallows. Check the right lobe of thyroid gland in the same way.

23. Palpate the position of the trachea.

24. From left to right, auscultate the cervical blood vessels. If the thyroid is not enlarged, there is no need to auscultate.

25. Test stiffness of the neck. With the patient lying down, remove the pillow and place the left hand under the patient’s occiput and place the right hand on their chest. Passively flex the patient’s neck and observe any movement of the knee and hip joints (Brudzinski’s sign).

**D. ANTERIOR CHEST AND LUNGS**

26. Inspect the skin of anterior chest, respiratory movement, intercostal spaces, and chest wall veins; squat down to observe the shape of the thoracic cage; inspect the positions of both breasts and nipples.

27. Palpate axillary lymph nodes. When palpating the left axillary lymph nodes, use your left hand to support the patient’s left forearm and put your right palm side close to the chest wall with the fingers together, palpating upward towards the axilla. Then palpate the posterior axillary wall, the medial axillary wall, and the anterior axillary wall. When palpating the anterior axillary wall, pay attention to the arrangement of the thumb and four fingers. Then, turning the palm outward, palpate the lateral axillary wall. Examine the right axillary lymph nodes in the same manner using the opposite hand.

28. Palpate and press the thoracic wall to measure the elasticity. Check for subcutaneous emphysema, chest wall tenderness and sternal tenderness.

When examining a female patient’s breasts, first compare one side with the contralateral side. From shallow to deep, palpate the breasts using the following sequence: superomedial, superolateral, tail, inferomedial, inferolateral. Finally, palpate the nipples.

29. Examine thoracic expansion. Place two palms with outstretched fingers on the lower anterior thorax bilaterally, with both thumbs along the sides of the costal margin pointing to the xiphoid process; the distance between the two thumbs should be about 2cm. Then ask the patient to take a deep breath.

30. Check vocal fremitus. Place two palms symmetrically in three positions (upper, middle and lower) on the patient’s chest, and ask the patient to say a prolonged "Yi" with sustained intensity, while moving hand positions between the three positions.

31. Check the pleural membrane movement. Place two palms symmetrically on the lower sides of the thorax, and ask the patient to breathe in deeply.

32. Check the distribution of resonance using chest percussion. Following the principles of percussion (lateral to medial, top to bottom, and comparing both sides), percuss from the first intercostal space to the fourth intercostal space.

33. Percuss the lower lung border. Percuss, in order, along the right midclavicular line, the left midaxillary line, and the right midaxillary line. Ask the patient to breathe normally. Percuss from top to bottom, and when the sound changes from resonant to dull, make a mark on the patient at the center part of your middle finger using a pen.

34. Lung auscultation. Ask the patient to breathe deeply. Auscultate the lungs along the midclavicular line, the anterior axillary line and the midaxillary line. Auscultate the lungs in three positions (upper, middle, and lower) bilaterally and symmetrically.

35. Check vocal resonance. Ask the patient to say a prolonged “Yi” sound with the same intensity. Auscultate bilaterally in three positions, (upper, middle, and lower), making sure to compare both sides.

36. Auscultate for pleural friction rubs. Ask the patient to breathe in deeply and auscultate in the anteroinferior lateral chest area.

**E. HEART**

37. Observe the precordium for any bulging and for the apical beat. The examiner squats and views tangentially to the patient to inspect for any abnormal precordial movement.

38. Palpate apical impulse, abnormal precordial pulsation (including subxiphoid pulsations) and thrills. With the palm on the precordium and apex of the heart, use the palm (hypothenar eminence) to confirm the location and periodicity of the apical beat.

39. Palpate for any pericardial friction rubs. Place the palm along the left sternal border on the third and fourth intercostal space.

40. Percuss the borders of the heart. Begin percussing on the left side of the chest, 2~3cm lateral to the apical impulse, and move medially until resonance changes to dullness, then mark the margin of dullness. Percuss in this way up to the second intercostal space. Then percuss the right heart border along the right midclavicular line, moving from superior to inferior and lateral to medial until cardiac dullness is appreciated, and from inferior to superior to the second intercostal space. Finally, using a ruler, measure the vertical distance of the marked cardiac borders to the midline and the distance between the midline and the left midclavicular line.

41. Cardiac auscultation. First place the diaphragm on the point of maximal impulse. Auscultate for heart rate (1 min), heart rhythm, heart sounds (changes in intensity, splitting of heart sounds, and extra heart sounds.), and murmurs. Then use the following sequence to auscultate the remainder of the heart: Pulmonic area→Aortic area→2nd Aortic area→Tricuspid area.

42. Auscultate for pericardial friction rubs. Listen along the left sternal border in the third and fourth intercostal spaces.

**Sit up, and check the back!**

**F. BACK**

43. Inspect the skin with the patient seated with their hands on their knees, exposing the back.

44. Palpate chest wall expansion. From behind the patient, place both hands symmetrically on the back, with the thumbs at the 10^th^ costal level 2 cm apart, hands pushing towards the direction of the spine, the palms causing laxity of the skin, and have the patient breathe in and out deeply.

45. Palpate vocal fremitus. Place both palms symmetrically on the infrascapular region of the back, and ask the patient to say the prolonged sound “Yi”. Cross the hands over and ask the patient to repeat the sound.

46. Percussion of the back. Four areas of the back in the area of the spine between the scapular regions, and four areas along the posterior axillary line and scapular line, bilaterally.

47. Percuss the lower lung border and its range of movement. Ask the patient to breathe normally and percuss the let lower lung border from superior to inferior along the left scapular line. Then ask the patient to take deep breath in and hold it for a moment, and quickly percuss from superior to inferior, noting where resonance changes to dullness, and making a mark in the center part of the middle finger at that location. Finally ask the patient to take deep breath out and hold, and percuss from superior to inferior quickly, noting where resonance changes to dullness and marking that location. Have the patient resume normal breathing and measure the distance between the two marks. Percuss the right lung border and its range of movement in the same manner.

48. Auscultation of the back. Four areas of the back in the area of the spine between the scapular regions, and four areas along the posterior axillary line and scapular line, bilaterally.

49. Auscultate vocal resonance. Place the stethoscope bilaterally and symmetrically in a total of four places on the back: on both sides of the spine in the interscapular region and infrascapular regions. Ask the patient to say prolonged “Yi” sound . Compare to the opposite side.

50. Examine the lower costal margin and check for any costovertrebal angle tenderness bilaterally.

51. Observe spinal mobility.

52. Observe the degree of curvature of the spine, and check for tenderness to palpation or percussion of the spine (first using indirect percussion, then by direct percussion).

**Supine, continue to examine the abdomen!**

**G. ABDOMEN**

53. Inspect the abdominal contour (squat down and observe horizontally), abdominal skin, respiratory movement, abdominal varicosities, and gastrointestinal or peristaltic waves.

54. Auscultate for bowel sounds in the right lower quadrant of the abdomen (one minute).

55. Auscultate for vascular murmurs.

56. Check the distribution of abdominal percussion sounds. Beginning with the left lower quadrant, percuss the four abdominal quadrants in counterclockwise order.

57. Percuss the upper and lower liver margins.

58. Check for pain to percussion in the liver.

59. Percuss for shifting dullness. Beginning with the periumbilical region, percuss moving towards the left along the umbilical level until dullness is perceived. Then ask the patient to lie on his right side, and percuss the area again, moving to the right and stopping in the area of dullness. Ask the patient to lie on his left side, 180 degrees, in the left lateral decubitus position, and wait a moment before percussing again.

60. Superficial abdominal palpation. Begin with the left lower quadrant, palpating shallowly all areas of the abdomen in counterclockwise order. Check for rebound pain at McBurney’s point.

61. Deep abdominal palpation. Using both hands (overlapping), palpate the deeper abdominal organs using the fingertips. Begin with the left lower quadrant and palpate in a counterclockwise direction.

62. Palpate the liver. Place the left thumb on the lower rib cage and the remaining four fingers on the back. Place the right hand on the right iliac fossa and palpate upward along the midclavicular line to the costal margin until the hepatic border or costal margin is reached. If liver is reached, percuss the upper border of the liver along the midclavicular line and measure the distance from the upper and lower borders to calculate the size of the liver. Check for hepatojugular reflux in patients with hepatomegaly.

63. Palpate the liver at the body midline. Generally, begin palpating from the umbilicus and move superiorly along the midline, pay attention to match the patient’s breathing. Then measure the distance between the liver edge and the xiphoid.

64. Check for tenderness of the gallbladder and Murphy's sign. Press in the left thumb at the junction of the lateral border of the rectus abdominis muscle and the arch of rib, and place the remaining four fingers across the ribs. Ask the patient to take a deep breath in, and pay attention to the patient’s facial expressions and response which indicate discomfort and pain.

65. Palpate the spleen. Place the left hand behind the left rib cage, from about 7th to the 10th rib, and with the right hand begin palpating the spleen starting from the umbilicus. Try to move with the patient’s breathing, and glide more deeply towards the arch of rib until you touch the splenic margin or left costal margin. If the spleen is not palpated, have the patient roll onto his right side (keeping the right leg straight and left leg flexed), and palpate again. In patients with splenomegaly, measure the distance of the Jia Yi line, Jia Bing line, and Ding Wu line.

66. Palpate kidneys using the bimanual method.

67. Check for a fluid thrill. Place the left hand lightly on the right side of the abdominal wall, and percuss the left abdominal wall using the fingers of the right hand. If necessary, obtain the help of the patient or an assistant and have him place the ulnar side of the palm on the abdominal midline, then percuss the opposite side of the abdominal wall.

68. Check for splashing sound. Place the left ear close to the upper abdomen of the patient and gently strike the upper abdomen.

(According to the professional standards, the abdominal examination follows the following sequence: Inspection--- Auscultation---Palpation---Percussion).

69. Palpate the inguinal lymph nodes and femoral pulses in the inguinal regions.

70. Auscultate for the “pistol shot” and Duroziez’s double murmur by placing the stethoscope on the femoral artery.

71. Check the upper, middle, and lower abdominal reflexes bilaterally.

**H. EXTREMITIES AND REFLEXES**

72. Inspect the skin, joints, fingers and nails of bilateral upper limbs.

73. Check skin elasticity in the medial area 3-4 cm above the elbow. Palpate bilateral supratrochlear lymph nodes.

74. Palpate bilateral radial artery pulses, evaluating for pulsus alternans, pulsus paradoxus, water-hammer pulse, and capillary pulse. When checking for a water-hammer pulse, use the left hand palmar side to grasp the patient’s right radial artery and raise the patient’s forearm over his head.

75. Evaluate the range of motion and muscle strength of bilateral upper extremities.

76. From left to right, check the biceps reflex, triceps reflex, brachioradial reflex, and Hoffmann's sign.

77. Inspect the skin, veins, joints, ankles, and nails of bilateral lower extremities.

78. From left lower extremity to right lower extremity, palpate the popliteal lymph nodes and check for pitting edema. Palpate bilateral dorsalis pedis pulses.

79. Evaluate the motor function and muscle strength of bilateral lower limbs.

80. Check the patellar reflex, achilles tendon reflex, Babinski sign, Copperhead sign, Gordon's sign, and Kernig's sign on both sides.

81. Place the patient’s blanket on him, tidy up any instruments. Thank the patient for their cooperation, and say goodbye.
