# Supplementary material for: Performance of Physical Examination Skills in Medical Students during Diagnostic Medicine Course in a University Hospital of Northwest China
Source: PLoS One. 2014 Oct 15;9(10):e109294. doi: 10.1371/journal.pone.0109294 (PMC4198092; doi:10.1371/journal.pone.0109294)
Supplement: Table S2 — Evaluation checklist of students' PE skills. (DOC) [file pone.0109294.s002.doc]

**Table S2** Evaluation checklist of students’ PE skills

| Student ID | Name | Gender | Assessment  content | Communication and care skills | | Items | | Appropriateness of examination sequence | | Examination techniques | | Time taken | |
| --- | --- | --- | --- | --- | --- | --- | --- | --- | --- | --- | --- | --- | --- |
|  |  |  |  | Right or  Wrong | Details | Right or  Wrong | Details | Right or  Wrong | Details | Right or  Wrong | Details | Right or  Wrong | Details |
|  |  |  |  |  |  |  |  |  |  |  |  |  |  |
|  |  |  |  |  |  |  |  |  |  |  |  |  |  |
