# Supplementary material for: Performance of Physical Examination Skills in Medical Students during Diagnostic Medicine Course in a University Hospital of Northwest China
Source: PLoS One. 2014 Oct 15;9(10):e109294. doi: 10.1371/journal.pone.0109294 (PMC4198092; doi:10.1371/journal.pone.0109294)
Supplement: Table S3 — Demographics and source regions of the medical students enrolled in 2008 in three universities of China. (DOC) [file pone.0109294.s003.doc]

**Table S3 Demographics and source regions of the medical students enrolled in 2008 in three universities of China**

|  | Xi'an Jiaotong University  (n = 72) | Harbin Medical University  (n = 114) | Anhui Medical University  (n = 210) | *p* |
| --- | --- | --- | --- | --- |
| Gender (M/F)* | 32/40 | 38/76 | 127/83 | < 0.001 |
| Age [years, range (mean ± SD)] | 19-25 (21.2±1.3) | 19-25 (21.7±1.0) | 19-25 (21.5±1.4) | 0.876 |
| Ethnic |  |  |  | 0.132 |
| Minority | 2 (2.8%) | 6 (5.3%) | 3 (1.4%) | - |
| Han | 70 (97.2%) | 108 (94.7%) | 207 (98.6%) | - |
| Language | Chinese | Chinese | Chinese | - |
| Source regions** |  |  |  |  |
| Number | 18 | 17 | 5 | - |
| Province | Shaanxi, Xingjiang, Shandong, Xinjiang, Zhejiang, Henan, Hubei, Jiangsu, Anhui, Beijing, Gansu, Guangxi, Hebei, Ningxia, Hunan, Sichuan, Shanxi, Yunnan, Chongqing | Heilongjiang, Beijing, Fujian, Guangdong, Guangxi, Hebei, Henan, Jilin, Jiangsu, Jiangxi, Shandong, Shanxi, Sichuan, Tianjin, Yunnan, Zhejiang, Chongqing | Anhui, Fujian, Shandong, Zhejiang, Jiangsu |  |

* Gender: Xi'an Jiao Tong University vs. Harbin Medical University *p*=0.128; Xi'an Jiao Tong University vs. Anhui Medical University *p*=0.175; Harbin Medical University vs. Anhui Medical University *p* <0.001. ** Provinces, Municipalities directly under the central government, and Autonomous regions.
